# Combining enyne metathesis with long-established organic transformations: a powerful strategy for the sustainable synthesis of bioactive molecules

**DOI:** 10.3762/bjoc.16.68

**Published:** 2020-04-16

**Authors:** Valerian Dragutan, Ileana Dragutan, Albert Demonceau, Lionel Delaude

**Affiliations:** 1 Institute of Organic Chemistry of the Romanian Academy, Bucharest, 060023, Romania; 2 Laboratory of Catalysis, Institut de Chimie (B6a), Allée du six Août 13, Université de Liège, 4000 Liège, Belgium

**Keywords:** bioactive compounds, enyne metathesis, ring-closing metathesis, ruthenium catalysts, tandem reactions

## Abstract

This account surveys the current progress on the application of intra- and intermolecular enyne metathesis as main key steps in the synthesis of challenging structural motifs and stereochemistries found in bioactive compounds. Special emphasis is placed on ruthenium catalysts as promoters of enyne metathesis to build the desired 1,3-dienic units. The advantageous association of this approach with name reactions like Grignard, Wittig, Diels–Alder, Suzuki–Miyaura, Heck cross-coupling, etc. is illustrated. Examples unveil the generality of such tandem reactions in providing not only the intricate structures of known, in vivo effective substances but also for designing chemically modified analogs as valid alternatives for further therapeutic agents.

## Introduction

Alkene and alkyne metathesis [1–4], constituting highly versatile and powerful catalytic processes for constructing complex organic molecules [5–12], have found broad application in the fields of pharmaceutical synthesis [13–15], materials science [16–20], or in advanced techniques and technologies [21–30]. Thus, numerous multistep total syntheses of organic compounds, including bioactive molecules [31–35] and natural products [36–37], have been performed in a highly chemo- and stereoselective manner through metathesis routes [38–43]. In ingeniously elaborated procedures, olefin metathesis has been frequently employed as such or associated with name reactions like Grignard, Wittig, Diels–Alder, Suzuki–Miyaura, Heck, etc., resulting in the assembly of diverse intricate building blocks of the targeted structures [44]. Among the various embodiments of olefin metathesis, the highly chemoselective enyne metathesis reaction [45–49] has led to some of the most striking advances in the development of modern, efficient synthetic protocols [50–51]. Thus, the present account focuses on the impressive potential of enyne metathesis in providing sustainable access to bioactive organic compounds, when used in conjunction with a number of name reactions.

**Scheme 1:**
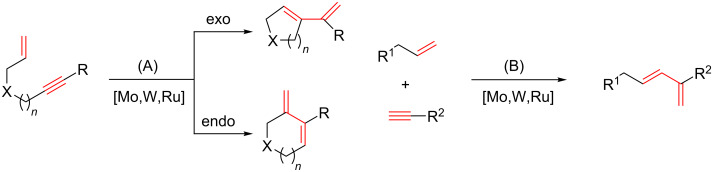
Intramolecular (A) and intermolecular (B) enyne metathesis reactions.

Enyne metathesis is a fundamental chemical transformation involving an alkene and an alkyne to produce a dienic structure through unsaturated bond reorganization [45–46]. This process may follow an intra- or intermolecular course (Scheme 1, A or B). The reaction is promoted by an array of metathesis catalytic systems, particularly derived from Mo, W, or Ru carbenes or it is induced by specific conventional transition-metal salts.

**Scheme 2:**
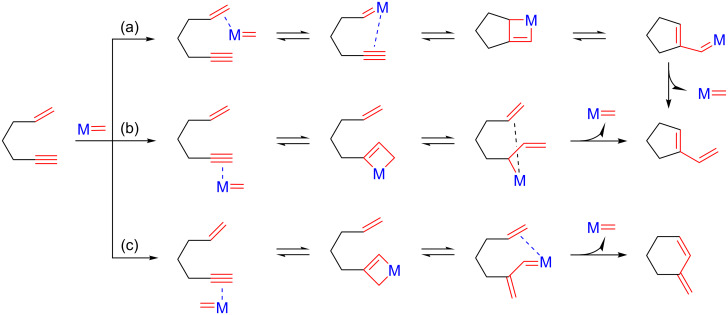
Ene–yne and yne–ene mechanisms for intramolecular enyne metathesis reactions.

It should be pointed out that enyne metatheses are atom economical processes driven by the enthalpic stability of the conjugated diene products. Depending on the steric requirements of the transition metal carbene and of the starting enyne, the intramolecular reaction can proceed either via ene–yne or yne–ene pathways to yield the respective dienic compounds (Scheme 2) [52].

**Scheme 3:**
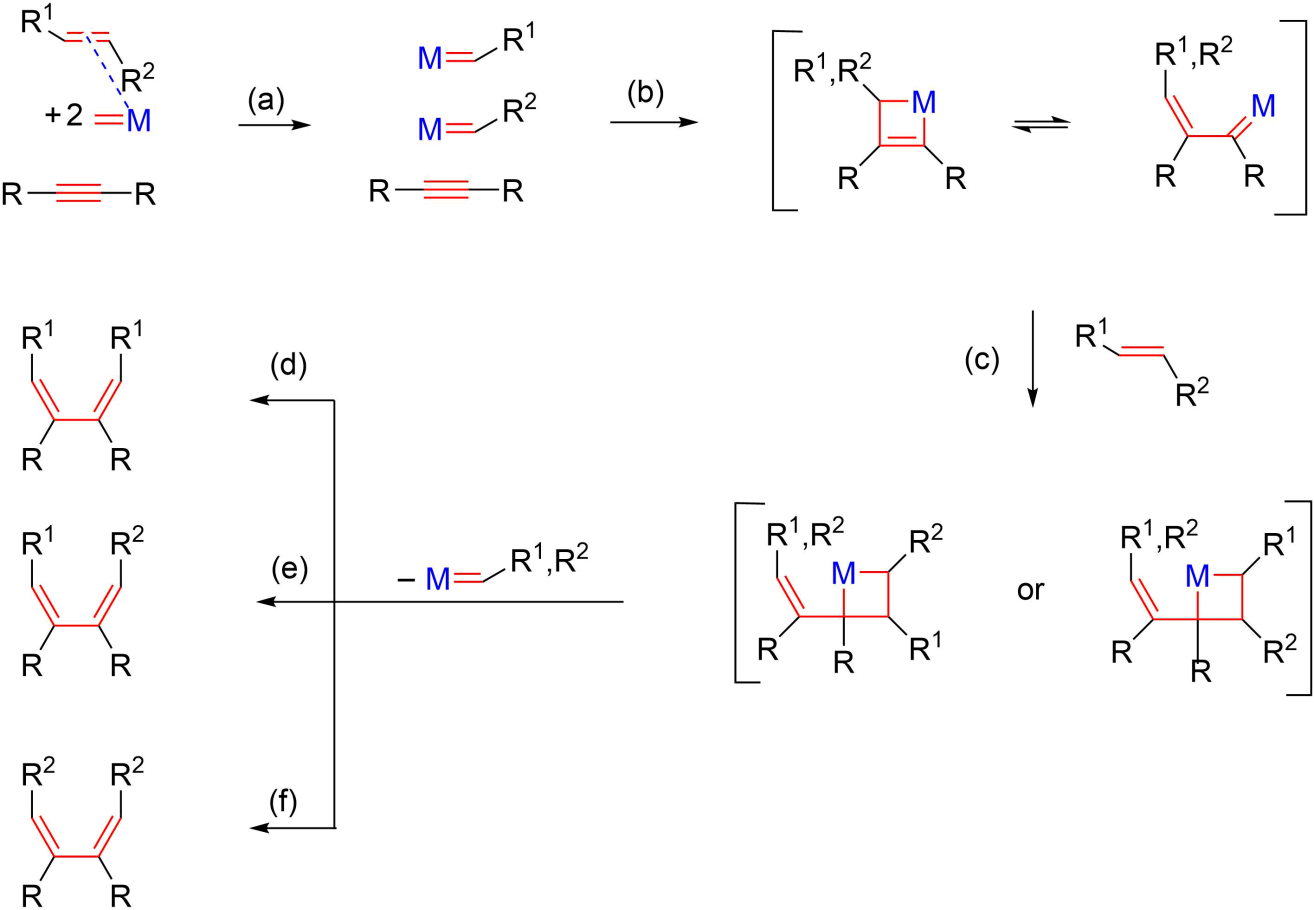
Metallacarbene mechanism in intermolecular enyne metathesis.

The reaction course is essential for directing the process towards the desired *exo* or *endo* isomer, in compliance with the required stereochemistry of the final product. For the intermolecular enyne metathesis reaction, the double bond of the alkene is coordinated to the metallacarbene and cleaved (Scheme 3 (a)) and the formed alkylidene species is inserted into the alkyne unit through a metallacyclobutene intermediate. This metallacyclobutene, through the rearrangement to a vinyl metal-alkylidene (Scheme 3 (b)) and subsequent metathesis with the alkene (Scheme 3 (c)) leads to the expected 1,3-diene (Scheme 3 (d–f)).

A particular advantage of the enyne metathesis is that the stereoselectivity can be readily controlled by the intramolecular vs the intermolecular process. Among the vast array of bioactive organic molecules already synthesized through enyne metathesis as a key step, we herein survey representative examples where this reaction is effectively associated with traditional chemical transformations to produce bioactive synthetic targets.

## Review

### Artemisinin and nanolobatolide

**Scheme 4:**
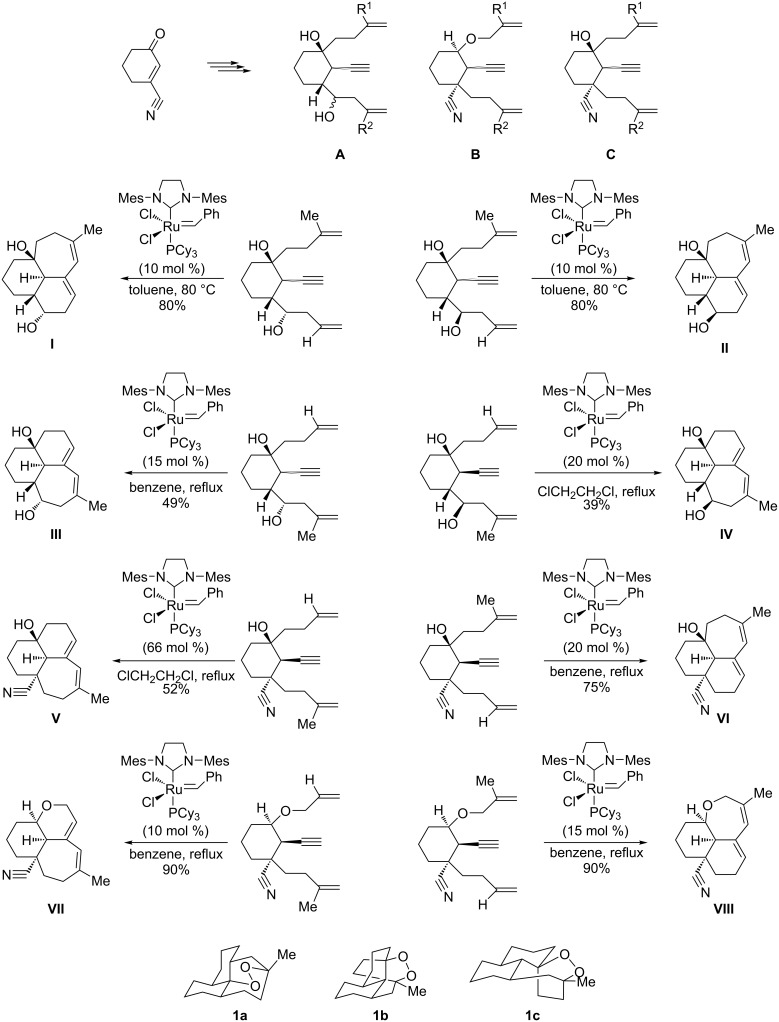
The Oguri strategy for accessing artemisinin analogs **1a–c** through enyne metathesis.

Due to its biological relevance, artemisinin, a tricyclic compound bearing a peroxide bridge, has been subject of extensive scientific investigations during the last decade [53–63]. In this context, Seeberger and co-workers successfully developed an ingenious continuous-flow process for the hemisynthesis of pure artemisinin from dihydroartemisinic acid (DHAA) [64–65]. Also, high efficient and recyclable catalytic systems based on metal-organic frameworks (MOFs) have also been reported for the tandem hemisynthesis of artemisinin [66]. In a remarkable work, this antimalarial drug was obtained by a new route involving enyne metathesis as the key step, adding this protocol to the existing synthetic or hemisynthetic procedures. Along this line, Oguri et al. elaborated an excellent strategy for assembling the tricyclic diene scaffold of artemisinin and its analogs that combines classical transformations with tandem dienyne ring-closing metathesis (RCM) [67–68]. Starting from 3-cyanocyclohex-2-enone, the authors obtained a versatile intermediate able to provide the appropriate dienyne precursors (**A**–**C**, Scheme 4) by multicomponent Grignard addition-alkylations. Through divergent cyclizations involving a chemoselective enyne metathesis catalyzed by Grubbs 2nd generation Ru-carbene, these intermediates then led to the tricyclic sesquiterpenoid-like scaffolds **I–VIII** (Scheme 4), as suitable precursors for the synthesis of artemisinin and its analogs (**1a–c**). After selecting the optimal tricyclic intermediate, the installation of the bridged peroxide groups in the corresponding artemisinin analog was eventually achieved by applying further oxidation.

**Scheme 5:**
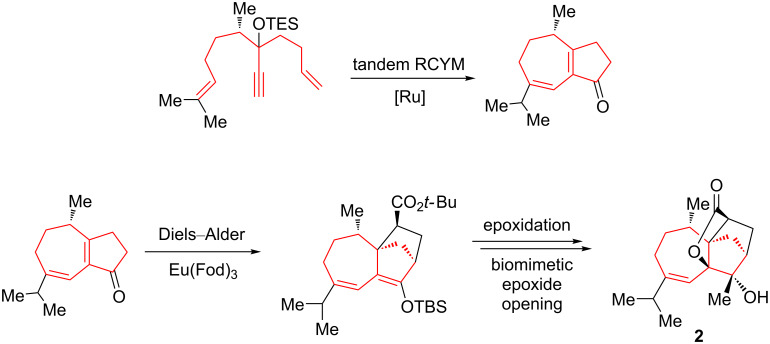
Access to the tetracyclic core of nanolobatolide (**2**) via tandem enyne metathesis followed by an Eu(fod)_3_-catalyzed intermolecular Diels–Alder cycloaddition, an epoxidation, and a biomimetic epoxide opening.

In a similar way to the above Oguri protocol, Li et al. prepared nanolobatolide (**2**), a potent neuroprotective agent, by successfully applying a tandem ring-closing metathesis of dienynes and subsequent Eu(fod)_3_-catalyzed intermolecular Diels–Alder cycloaddition and epoxidation reactions (Scheme 5) [69]. In this stereoselective synthesis, the last biomimetic step was critical to obtain the proper enantiomer of the tetracyclic core of nanolobatolide.

### Amphidinolide macrolides

**Scheme 6:**
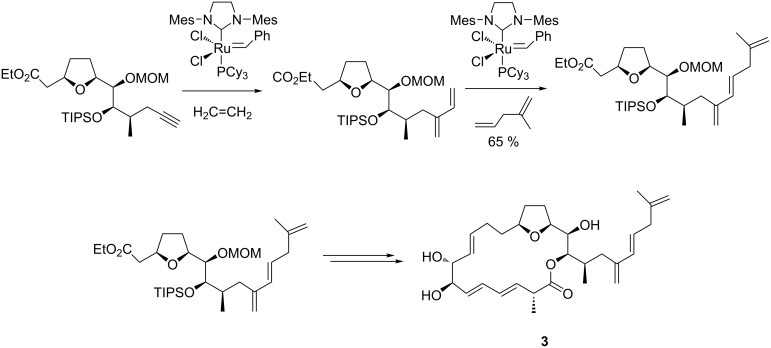
Synthesis of (−)-amphidinolide E (**3**) using an intermolecular enyne metathesis as the key step.

Amphidinolides constitute a broad family of natural macrolides that act as powerful cytotoxic agents against various cancer cell lines. Due to the stereochemical intricacy and high cytotoxicity, these compounds have attracted a great deal of attention from synthetic chemists. The application of an intermolecular enyne metathesis in tandem with a diene cross-metathesis as the crucial steps were reported by Lee in the total synthesis of (−)-amphidinolide E (**3**) (Scheme 6) [70]. It is noteworthy that the second-generation Grubbs catalyst was quite active and stereoselective for both the enyne and cross-metathesis steps, thereby affording the triene intermediate in a substantial yield. Additional transformations using conventional methods efficiently provided (−)-amphidinolide E (**3**).

**Scheme 7:**
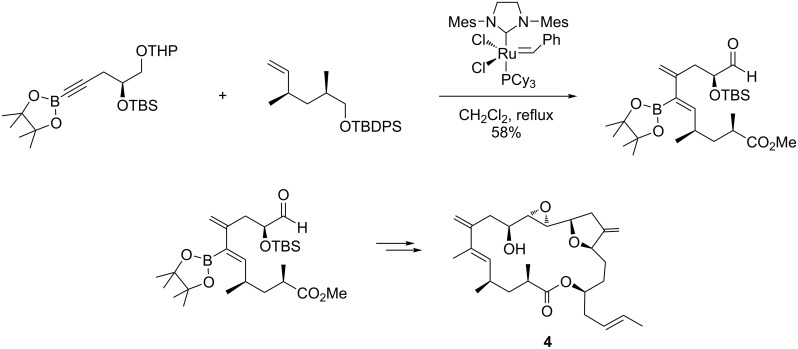
Synthesis of amphidinolide K (**4**) by an enyne metathesis route.

Amphidinolide K, another important bioactive macrolide endowed with specific cytotoxic activity against L1210 and KB cancer cells, was also obtained by Lee who carried out an intermolecular enyne metathesis between an alkynyl boronate and an olefinic substrate in the presence of the Grubbs 2nd generation catalyst [71]. The reaction was highly stereoselective and favored the formation of the desired *E-*isomer (*E/Z* = 7.5:1). The functionalization of the dienic compound through a Suzuki–Miyaura coupling and Julia–Kocienski olefination, followed by a Yamaguchi lactonization, and an asymmetric epoxidation in the presence of (+)-diethyl tartrate, conveniently produced (−)-amphidinolide K (**4**, Scheme 7).

**Scheme 8:**
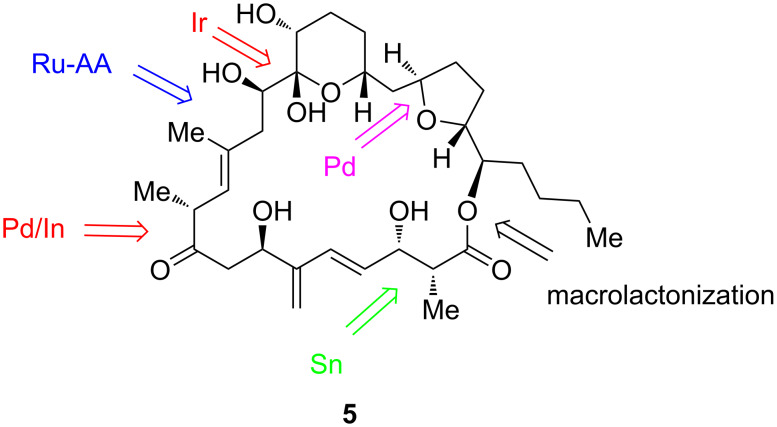
Trost synthesis of *des-epoxy*-amphidinolide N (**5**) [72].

In a remarkable work, Trost et al. [72] accomplished the convergent synthesis of *des*-epoxy-amphidinolide N (**5)** in 33 total steps (22 longest linear). To this end, after three generations of synthetic attempts, they succeeded joining the northern and southern fragments of *des*-epoxy-amphidinolide N, both endowed with a considerable level of structural complexity, by essentially using a Ru-catalyzed alkene–alkyne (Ru-AA) coupling reaction and a macrolactonization step (Scheme 8).

**Scheme 9:**
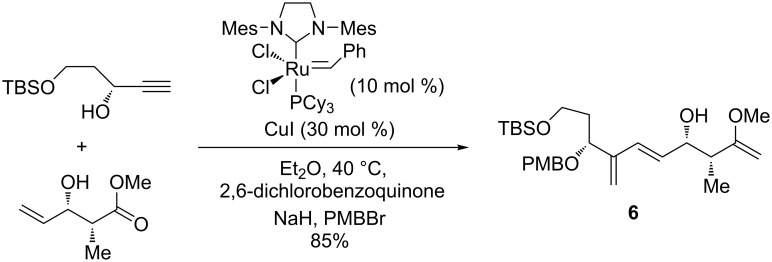
Enyne metathesis between the propargylic derivative and the allylic alcohol in the synthesis of the southern fragment of *des*-epoxy-amphidinolide N.

In this carefully designed total synthesis, after applying a Marshall coupling reaction aimed at installing a propargyl group into the initial acetylene component, an enyne metathesis between the formed propargylic derivative and an appropriate allylic alcohol promoted by the Grubbs second-generation catalyst, finally produced in high yield (85%) the intermediate diene **6**, as an essential building block for the southern fragment (Scheme 9).

**Scheme 10:**
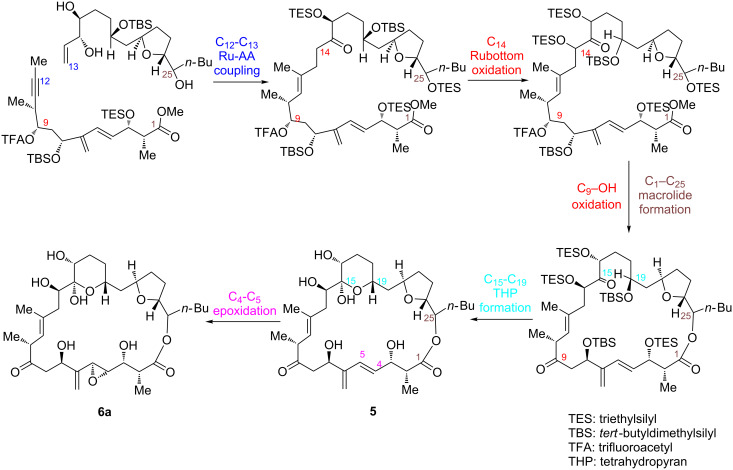
Synthetic route to amphidinolide N (**6a**).

Several other asymmetric transition-metal-catalyzed transformations were needed to achieve the total synthesis of *des*-epoxy-amphidinolide N, including a palladium asymmetric allylic alkylation (Pd-AAA), a Mukaiyama aldol reaction (with Sn), and a Krische allylation (with Ir) [72]. As special feature of this procedure, an Evans aldol reaction generated the *syn* aldol adduct, having the steric configuration imposed in *des*-epoxy-amphidinolide N. Also, in the last step of their work, Trost et al. managed to install the C_14_-OH via a finely tuned Rubottom oxidation that finalized the total synthesis of *des*-epoxy-amphidinolide N. Strikingly, an intramolecular Ru-catalyzed alkene-alkyne (Ru-AA) coupling and a late-stage epoxidation were readily accomplished, while the installation of the α,α′-dihydroxy ketone through a dihydroxylation proved difficult. Noteworthy, the structural elucidation of the THP ring of *des*-epoxy-amphidinolide N evidenced the hydrogen-bonding network of amphidinolide N (**6a**, Scheme 10).

Furthermore, a rigorous evaluation of the ^13^C NMR chemical shift differences suggested that amphidinolide N and its analog, carbenolide I, are identical chemical compounds [72].

**Scheme 11:**
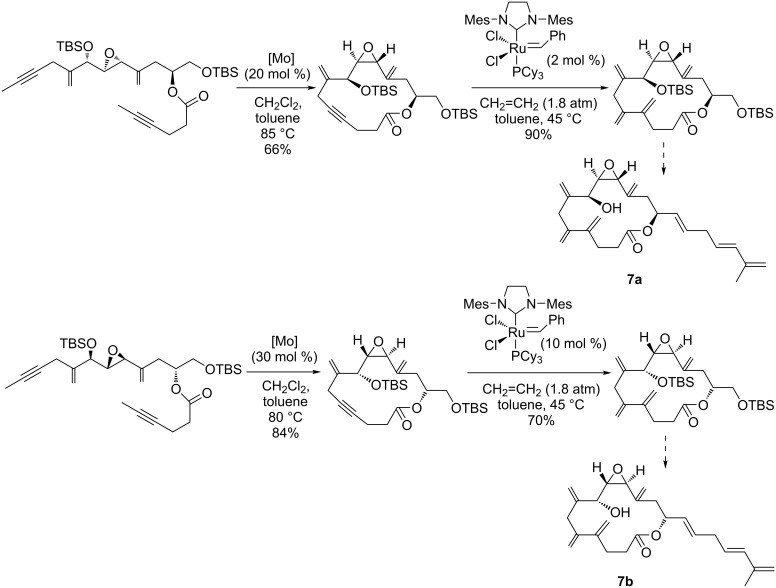
Synthesis of the stereoisomeric precursors of amphidinolide V (**7a** and **7b**) through alkyne ring-closing metathesis and enyne metathesis as the key steps.

In the course of their comprehensive studies on the total synthesis of amphidinolides [73–75], Fürstner et al*.* applied both ring-closing alkyne metathesis and intramolecular enyne metathesis with ethylene in a sequential mode for the synthesis of amphidinolide V (**7**) [75]. As a special merit of this original protocol, the macrocyclization of the stereoisomeric diyne precursors was performed first by a ring-closing alkyne metathesis in the presence of Schrock’s molybdenum catalyst. Next, the diene units were installed by intermolecular enyne metathesis of the preformed cyclic alkyne with ethylene using Grubbs second-generation ruthenium catalyst (Scheme 11). This innovative methodology allowed the installation of the vicinal *exo*‐methylene branches characteristic for the cytotoxic marine natural product amphidinolide V as well as the determination of its absolute configuration. It further enabled the synthesis of a set of diastereomers and analogs of amphidinolide V, whose biological activities were thoroughly evaluated. It is important to note, that the analogs of amphidinolide V designed by Fürstner gave the first insights into the structure–activity relationships for this family of compounds and revealed that the steric structure of the macrolactone is a highly critical parameter for their activity, while certain alterations of the side chain do not affect the cytotoxicity to a notable extent.

### Anthramycin

**Scheme 12:**
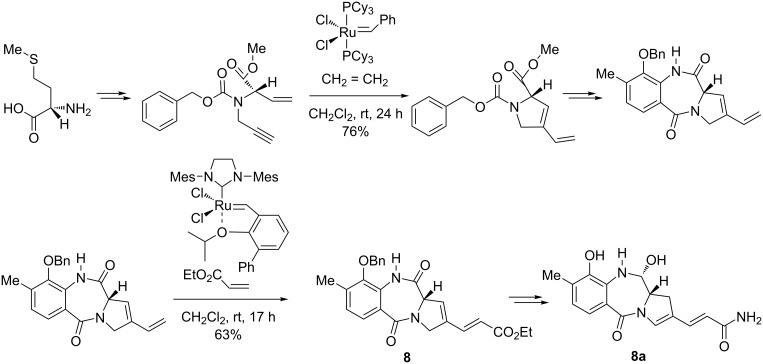
Synthesis of the anthramycin precursor **8** from ʟ-methionine by a tandem enyne metathesis–cross metathesis reaction.

In an interesting approach to the protected precursor **8** of (+)-anthramycin (**8a**), a compound with strong antitumor activity having a pyrrolobenzodiazepine structure, Mori et al. [76] successfully combined an enyne metathesis and an alkene cross-metathesis in an efficient sequential manner under the action of both the Grubbs first-generation and Hoveyda–Grubbs second-generation catalysts. According to this procedure, initially an enyne precursor was synthesized starting from ʟ-methionine. The subsequent enyne ring-closing metathesis in the presence of the Grubbs first-generation catalyst (5 mol %) afforded a pyrrolidine derivative in 76% yield (Scheme 12). This pyrrolidine was converted into a pyrrolo-1,4-benzodiazepinone bearing a vinyl side group, which underwent a cross-metathesis with ethyl acrylate in the presence of the Hoveyda–Grubbs second-generation-type catalyst (10 mol %) leading to the corresponding vinyl ester **8** in 63% yield, as a convenient precursor to (+)-anthramycin. The latter was obtained via an isomerization of the double bond in the pyrrolidine ring catalyzed by RhCl_3_·H_2_O, followed by debenzylation, amidation, and aminal generation using the Stille protocol [77].

### (–)‐Clavukerin A and related sesquiterpenes

**Scheme 13:**
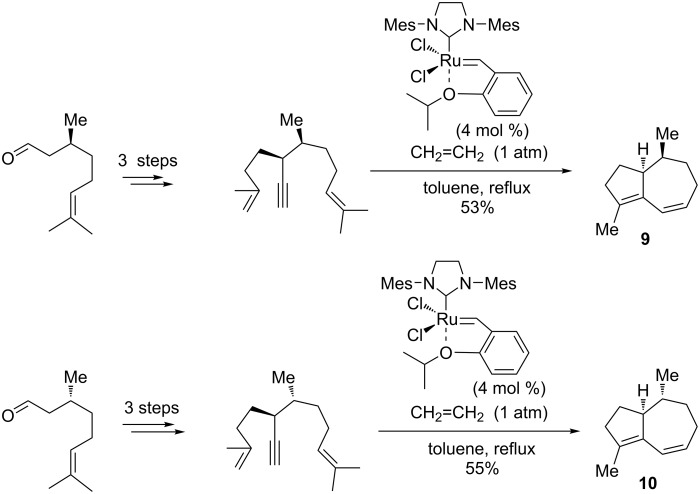
Synthesis of (−)‐clavukerin A (**9**) and (−)‐isoclavukerin A (**10**) by an enyne metathesis route starting from (*S*)- and (*R*)-citronellal.

Applying original organocatalytic/metal-catalyzed tactics, Metz et al. [78] reported a tandem dienyne RCM for the synthesis of the marine sesquiterpenoid (−)‐clavukerin A (**9**) and its stereoisomer (−)‐isoclavukerin A (**10**, Scheme 13). The sterically related analogs (+)-clavularin A and clavularin B have also been produced by this protocol. It is noteworthy, that the authors succeeded in preparing the two enantiopure dienyne precursors in three steps, from (*S*)- and (*R*)-citronellal, through a diastereoselective Michael addition, chemoselective dibromoolefination, and a one-pot Wittig olefination/alkyne-bond formation. The enantiopure dienynes were then converted into the enantiomeric hydroazulenes in 53% and 55% yield, respectively, by domino metathesis reactions using the Hoveyda–Grubbs catalyst (4 mol %). In the final step, (−)‐clavukerin A was effectively converted into (+)-clavularin A and the latter epimerized to (−)-clavularin B.

**Scheme 14:**
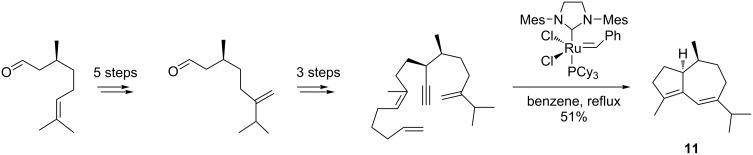
Synthesis of (−)-isoguaiene (**11**) through an enyne metathesis as the key step.

Recently, (–)-isoguaiene (**11**), a member of the guaiane sesquiterpenes and structurally related to the trinorsesquiterpene (−)‐clavukerin A, was also communicated by Metz et al. [79] using an enyne metathesis reaction as a key step. The authors performed a relay metathesis of the trienyne or diene–diyne precursors in the presence of the second-generation Grubbs catalyst (Scheme 14). The enantiopure trienyne or diene–diyne metathesis precursors were readily obtained from (*S*)-citronellal by a highly diastereoselective organocatalytic Michael addition.

### Erogorgiaene

**Scheme 15:**
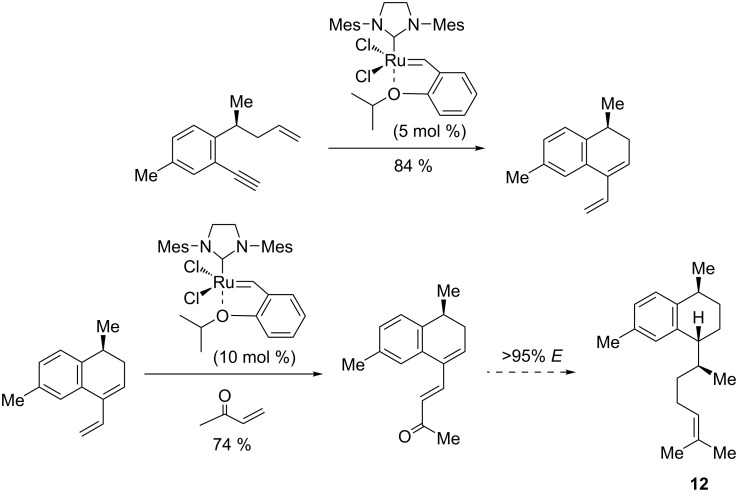
Synthesis of erogorgiaene (**12)** by a tandem enyne metathesis/cross metathesis sequence using the second-generation Hoveyda–Grubbs catalyst.

An exceptionally stereoselective synthesis of erogorgiaene (**12**) (95% *E*-selectivity) was reported by Hoveyda et al. [80]. They combined an enyne ring-closing metathesis and an alkene cross-metathesis reaction in a sequential mode, using the appropriate enantiopure enynes and the Hoveyda–Grubbs catalyst in both transformations (Scheme 15). High yields and excellent chemoselectivities toward the diene products were attained in both metathesis steps applying the second-generation Hoveyda–Grubbs Ru catalyst, while, unexpectedly, the second-generation Grubbs ruthenium catalyst was less active and gave rise to some side-products.

### (−)-Galanthamine

**Scheme 16:**
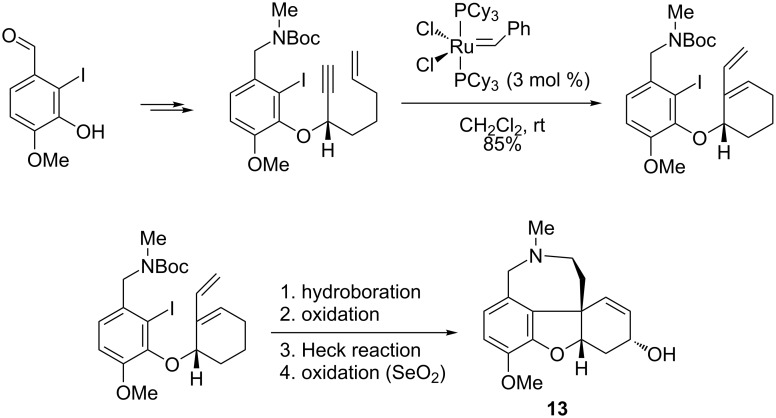
Synthesis of (−)-galanthamine (**13**) from isovanilin by an enyne metathesis.

An important alkaloid active in the treatment of mild to moderate Alzheimer's disease and other memory impairments, namely (−)-galanthamine (**13**), has attracted great interest for its structural complexity and specific bioactivity. The total synthesis of compound **13**, described by Brown et al. [81], involved an enyne ring-closing metathesis and a Heck cross-coupling as the key reactions for constructing the five-membered oxacyclic ring. As stated in this protocol, a functionalized enyne precursor was prepared in several steps with high enantioselectivity (92% ee), starting from isovanillin. This precursor underwent a ring-closing metathesis in the presence of the Grubbs first-generation Ru catalyst (3 mol %) to give the corresponding 1,3-diene intermediate in 85% yield (Scheme 16). The subsequent hydroboration and oxidation to homoallylic alcohol, followed by a palladium-catalyzed Heck cross-coupling, an allylic oxidation with SeO_2_, mesylation, and deprotection, afforded (−)-galanthamine (**13**) as the final product.

### Kempene diterpenes

**Scheme 17:**
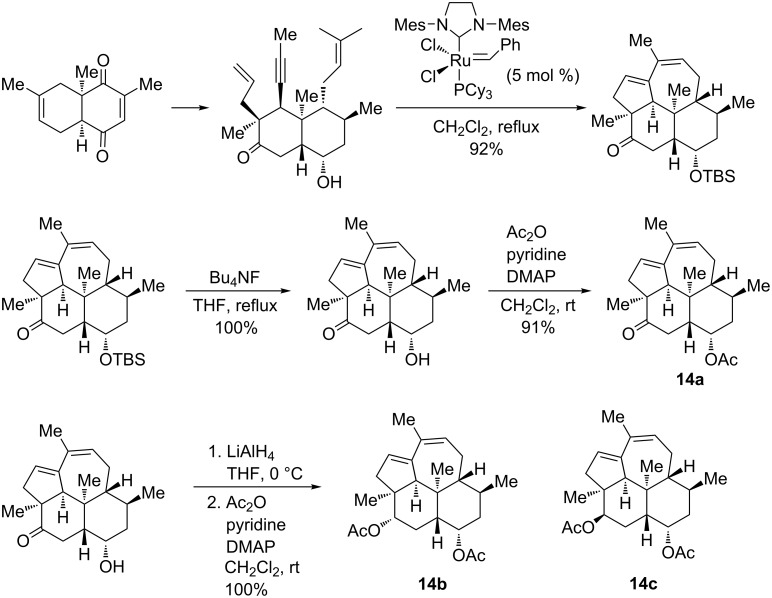
Application of enyne metathesis for the synthesis of kempene diterpenes **14a–c**.

The first enantioselective synthesis of kempene diterpenes **14a**–**c**, natural compounds exhibiting a significant antibiotic activity against *B. subtilis*, relying on the domino enyne metathesis of the adequate dienyne precursors as a key step, was disclosed by Metz et. al. [82]. The starting dienynes were obtained in a high enantiomeric purity starting form 2,6-dimethyl-1,4-benzoquinone and isoprene via an asymmetric Diels–Alder reaction. The domino metathesis reactions induced by the Grubbs second-generation catalyst proceeded in good yield (92%) thereby affording a protected tetracyclic kempane derivative. The latter was further converted into (+)-kempene-2 (**14a**) in 91% yield by deprotection and acetylation (Scheme 17). A reduction of the intermediate ketone with lithium aluminum hydride followed by an acetylation finally led to (+)-kempene-1 (**14b)** and (+)-3-*epi*-kempene-2 (**14c**). Importantly, the tandem metathesis of the dienyne precursor could also be performed in good yield (82%) with the Grubbs first-generation Ru catalyst (10 mol %).

### (+)-Lycoflexine alkaloid

**Scheme 18:**
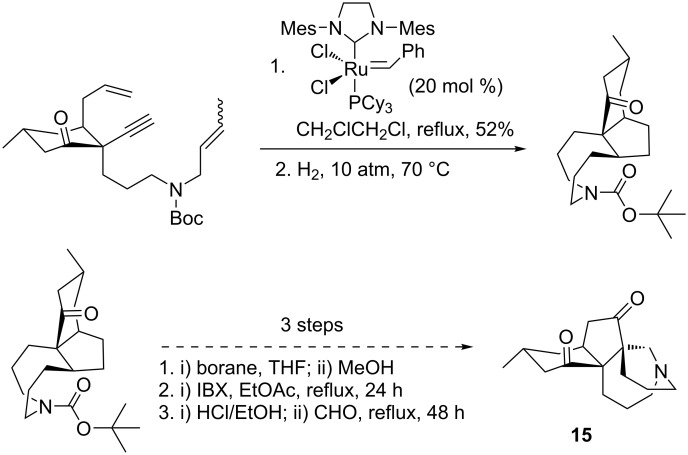
Synthesis of the alkaloid (+)-lycoflexine (**15**) through enyne metathesis.

In 2010, Ramharter and Mulzer [83] prepared an intricate tricyclic intermediate useful for the total synthesis of the alkaloid (+)-lycoflexine (**15**). In this strategy, first a protected dienyne precursor was prepared that, by an enyne tandem ring-closing metathesis induced by the Grubbs second-generation Ru catalyst, produced a tricyclic diene in 52% yield. Next, a selective hydrogenation of the *cis*-disubstituted double bond in the latter was performed under a high pressure of hydrogen, leading to the saturated tricyclic scaffold that was used as a key intermediate in the total synthesis of lycoflexine (**15**, Scheme 18).

### Manzamine alkaloids

**Scheme 19:**
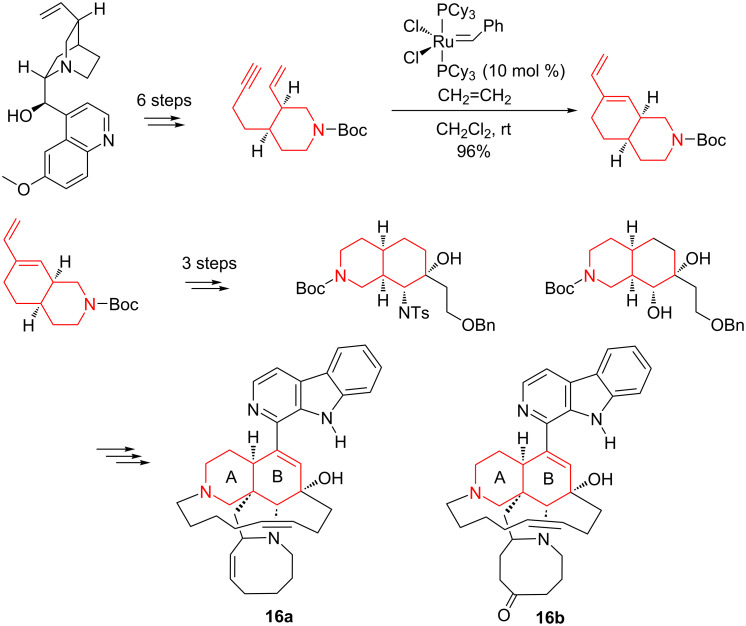
Synthesis of the AB subunits of manzamine A (**16a**) and E (**16b**) by enyne metathesis.

A highly interesting application of a metathesis reaction for accessing bioactive organic molecules is found in the complex synthesis of manzamine alkaloids. These efficient antitumor agents, originally isolated from several genera of marine sponges, contain a pentacyclic core with a pendant β-carboline moiety. Their total synthesis implies an elaborate assembly of the subunits leading to the pentacyclic scaffold. Previously, the AB ring system of manzamines was constructed mainly using Diels–Alder reactions [84–85]. Thus, Fukuyama et al. [85] described an elegant approach for the synthesis of (+)-manzamine A in a totally stereoselective manner relying on a Diels–Alder cycloaddition, an intramolecular Mitsunobu reaction, a [3,3]-sigmatropic rearrangement, and a ring-closing metathesis. As an alternative to this approach, Clark et al. [86] efficiently performed a sequential Ru-catalyzed enyne metathesis in combination with a hydroboration, and an aminohydroxylation. For this purpose, they first prepared the properly functionalized (–)-quinines from quinoline derivatives in six steps. These chiral intermediates were then submitted to an enyne metathesis reaction with the Grubbs first-generation Ru catalyst (10 mol %), under an ethylene atmosphere, to generate the corresponding bicyclic dienic scaffolds. The subsequent hydroboration and aminohydroxylation carried out on these bicyclic dienes provided the AB subunit as a key intermediate component of manzamines A (**16a**) and E (**16b**, Scheme 19). Eventually, several highly elaborated transformations of the AB subunit, including Diels–Alder cycloaddition and advanced functionalization reactions, gave access to manzamine A and E.

### Rhodexin A

**Scheme 20:**
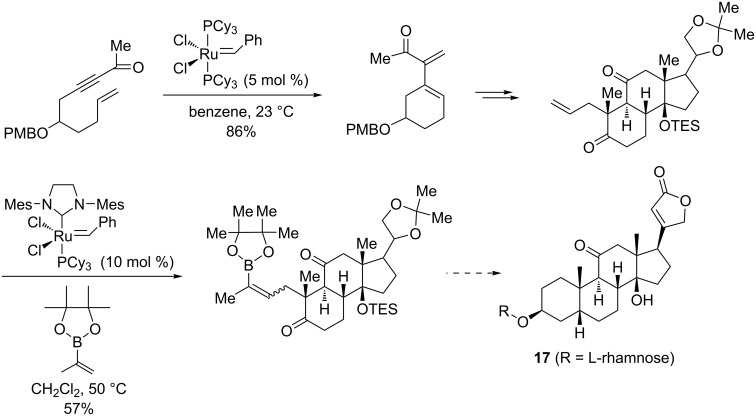
Jung's synthesis of rhodexin A (**17**) by enyne metathesis/cross metathesis reactions.

Jung et al. [87–89] succeeded in the stereoselective synthesis of rhodexin A (**17**), a steroid with potent cardiotonic properties and with activity against human leukemia K562 cells [87–88]. In this innovative work, the authors effectively combined an enyne metathesis promoted by the Grubbs first-generation catalyst and an alkene cross-metathesis induced by the Grubbs second-generation catalyst with reverse electron-demand Diels–Alder cycloadditions (Scheme 20). In contrast to the majority of steroids that are *trans*-fused, rhodexin A comprises *cis*-fused AB and CD rings. By carefully selecting the reaction conditions, they managed to impose this stereochemistry in all synthetic steps participating in the construction of the tetracyclic ring system of rhodexin A.

### Securinega alkaloids

**Scheme 21:**
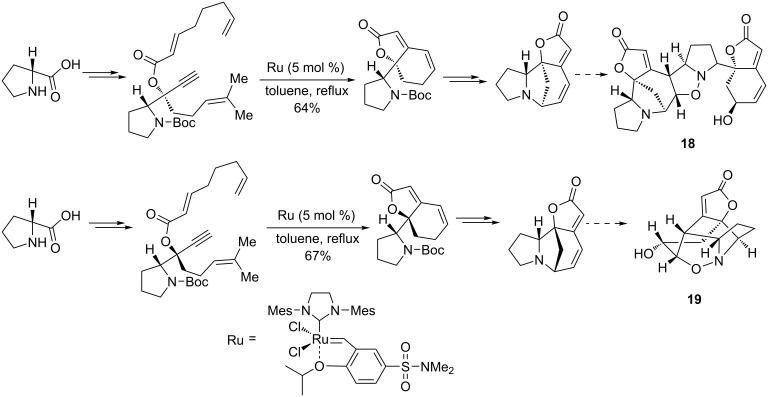
Total synthesis of (−)-flueggine A (**18**) and (+)-virosaine B (**19**) from Weinreb amide by enyne metathesis as the key step.

The total synthesis of the Securinega alkaloids, (−)-flueggine A (**18**) and (+)-virosaine B (**19**), which are potent anticancer agents, was proposed by Wei et al. [90] via a relay ring-closing metathesis (RRCM) associated with a 1,3-dipolar cycloaddition. The enyne precursors bearing a dienic relay unit, were prepared from Weinreb amide by an asymmetric pathway, were reacted with either the Grubbs second-generation catalyst, the Hoveyda–Grubbs second-generation catalyst, or the Zhan catalyst, the latter giving under the optimized conditions the highest yield of the expected dihydrobenzofuranones. Ultimately, (−)-flueggine A and (+)-virosaine B were obtained by 1,3-dipolar addition reactions (Scheme 21).

### Virgidivarine and virgiboidine

**Scheme 22:**
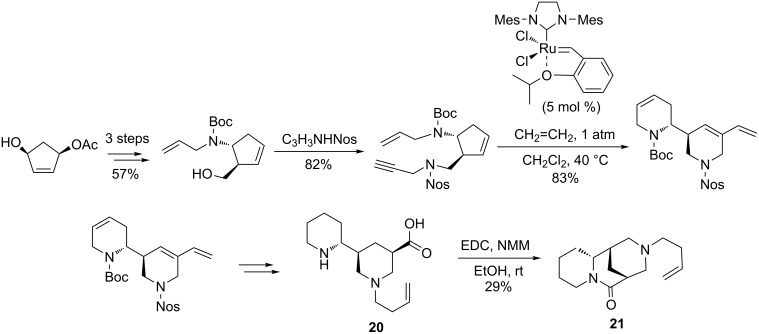
Access to virgidivarine (**20**) and virgiboidine (**21**) by an enyne metathesis route.

In 2013, Blechert et al. [91] devised an original methodology applying enyne metathesis as the key step for the total synthesis of virgidivarine (3-carboxy-*N*-(4'-butenyl)-5-(2'-piperidyl)piperidine) (**20)** and virgiboidine (3-but-3-en-1-yl)decahydro-6*H*-1,5-methanopyrido[1,2-*a*][1,5]diazocin-6-one) (**21**), two important pharmacologically active compounds containing dipiperidine and piperidino-quinolizidine units in their structure. Starting from the monoacetate of cyclopentene**-**1,4-diol that was obtained by the enzymatic desymmetrization of the corresponding diacetate, an enyne metathesis precursor was accessed by a Mitsunobu-type coupling reaction with propargylic amide. The ring-rearrangement metathesis (RRM) of this enyne precursor was carried out using the second-generation Hoveyda–Grubbs catalyst (5 mol %, 83% yield), under an ethylene atmosphere. The subsequent regioselective NaIO_4_-mediated oxidative cleavage of the pendant double bond, followed by the installation of the unsaturated *N*-butenyl group, oxidation, and deprotection provided the final products **20** and **21** (Scheme 22).

### (−)-Zenkequinone

**Scheme 23:**
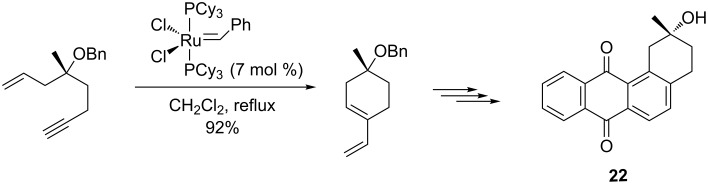
Enyne metathesis approach to (−)-zenkequinone B (**22**).

Also in 2013, Vangan and Kaliappan [92] disclosed an attractive protocol for the synthesis of (−)-zenkequinone B (**22**), a potent bioactive compound, by ring-closing enyne metathesis in the presence of the Grubbs first-generation Ru catalyst. According to this method, an enyne precursor was first converted into an exocyclic 1,3-diene in 92% yield. A Diels–Alder reaction with naphthoquinone and a deprotection step then led to the final compound (−)-zenkequinone B in a stereoselective manner (Scheme 23).

### *C*-Aryl glycosides

**Scheme 24:**
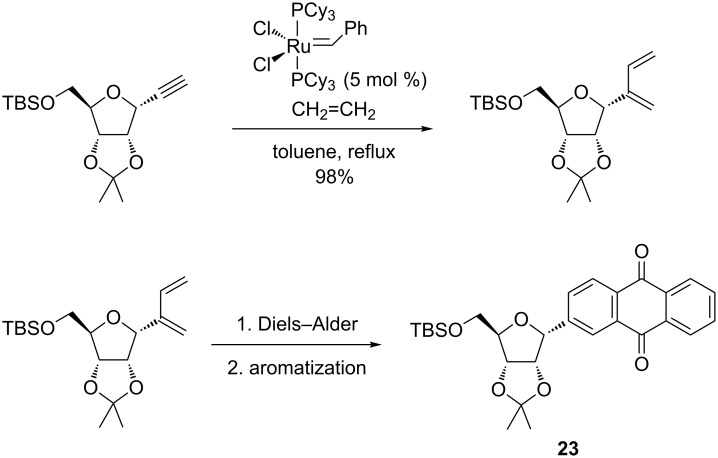
Access to *C*-aryl glycoside **23** by an intermolecular enyne metathesis/Diels–Alder cycloaddition.

**Scheme 25:**
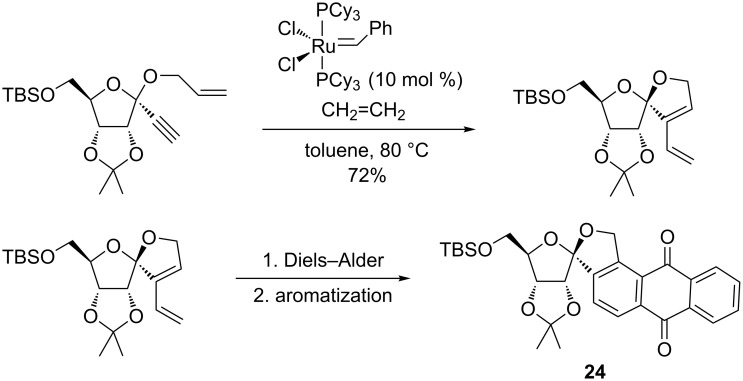
Synthesis of spiro-*C*-aryl glycoside **24** by a tandem intramolecular enyne metathesis/Diels–Alder reaction/aromatization.

Another interesting report from Kaliappan et al. [93] described a highly efficient synthesis of *C*-aryl glycosides, which are naturally occurring compounds of biological relevance. Starting from a glycoside precursor, the intermolecular enyne metathesis with ethylene gas in the presence of the second-generation Grubbs catalyst allowed the installation of the1,3-diene units at the anomeric center of the future *C*-aryl glycoside (**23**) in high yields (94–98%, Scheme 24). Alternatively, an intramolecular enyne metathesis promoted by the first-generation Grubbs catalyst produced the spiro-*C*-aryl glycoside **24** from sugar enyne precursors (Scheme 25). Subsequent Diels–Alder cycloaddition reactions with dienophiles and further aromatization reactions paved the way for a convenient access to structurally diverse polycyclic compounds. For instance, installing the *C*-aryl and spiro-*C*-aryl glycosides in the same moiety was successfully achieved. An application of this concept to obtain the core structure of gilvocarcin, a natural *C*-aryl glycoside, was also reported. Moreover, the authors attempted a tandem enyne metathesis/Diels–Alder/aromatization to directly prepare the *C*-aryl glycosides in a one-pot protocol.

### (−)-Exiguolide

**Scheme 26:**
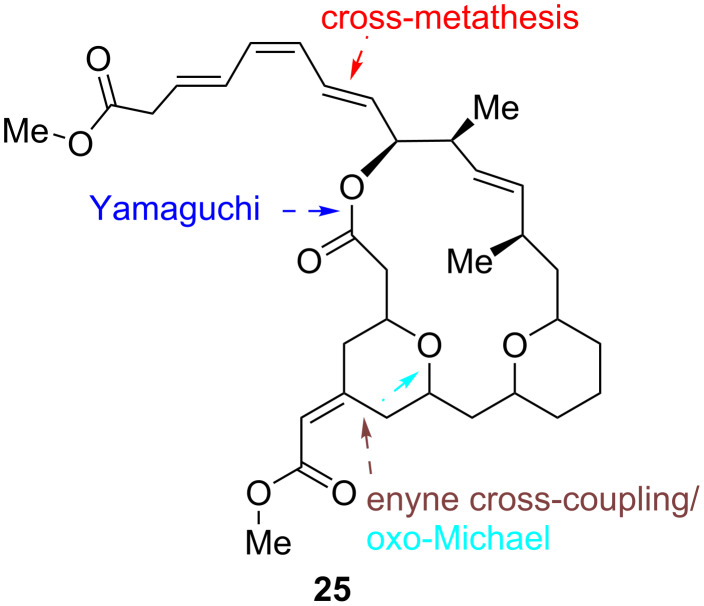
Pathways to (−)-exiguolide (**25**) by Trost’s Ru-catalyzed enyne cross-coupling and cross-metathesis [94].

The first stereoselective synthesis of the (−)-exiguolide enantiomer (**25**) was reported by Roulland et al. [94]. The method is a mechanistically distinct alternative to the enyne metathesis since it involves a Trost’s Ru-catalyzed enyne cross-coupling reaction associated with a Yamaguchi lactonization, a Grubbs Ru-catalyzed cross-metathesis, and a one-pot, two-step stereoselective conjugated allylic alcohol substitution (Scheme 26). It should be emphasized that in this convergent synthesis of (−)-exiguolide, the authors achieved a rigorous stereocontrol of both the exocyclic and endocyclic double bond geometries, as well as the stereoselective formation of the tetrahydrofuran rings.

## Conclusion

This review highlighted the most recent efforts regarding the development of enyne metathesis-based syntheses of complex bioactive, natural and nonnatural organic molecules. Both, intra- and intermolecular enyne metatheses have been valorized to efficiently produce key 1,3-dienic frameworks, further subjected to sequential functionalization. The main focus was placed on strategies combining enyne metathesis with traditional chemical transformations such as Diels–Alder cycloaddition, Suzuki–Miyaura or Heck cross-couplings, aromatization, epoxidation, hydroxylation, etc. This point of view has allowed to master a concise access to the target products which require exceptional chemical and stereochemical complexity. The excellence of the Grubbs- and Schrock-type metathesis catalysts as selective and proficient promoters of enyne metathesis was emphasized. The review also shed light on the continuous improvement of protocols relying on this reaction, potentially leading to tailorable properties of earmarked therapeutic agents.
